# Supplementary material for: Unexpected functional role of the transactivation domain for nuclear import of STAT5
Source: Commun Biol. 2026 Sep 26;9:1257. doi: 10.1038/s42003-026-10898-2 (PMC13615974; doi:10.1038/s42003-026-10898-2)
Supplement: Supplementary file 1 [file 42003_2026_10898_MOESM1_ESM.pdf]

nature portfolio

Peer Review File

Unexpected functional role of the transactivation domain for nuclear import of STAT5

Corresponding Author: Professor Gerhard Müller-Newen

This file contains all reviewer reports in order by version, followed by all author rebuttals in order by version.

Version 0:

Reviewer comments:

Reviewer #1

(Remarks to the Author)
Ernst et al present a well-executed and noteworthy study on STAT5 nuclear import. The authors demonstrate, using the appropriate controls, that the STAT5 transactivation domain (TAD) is required for the cytokine-induced nuclear accumulation of activated STAT5. In fact, they identify two key amino acids, and they demonstrate that nuclear-import assistance by the TAD is not required for other STAT proteins and cannot be provided by the TAD of another STAT.
The phenomenon manifests as accumulation of phosphorylated STAT5 (P-STAT5) in the cytoplasm upon cytokine treatment. To my knowledge, this is the first report of such behaviour for a full-length STAT protein; it is characterised thoroughly in this paper yet underlying molecular mechanisms (e.g. enhanced export and/or reduced import) remain opaque. Of note, the same phenomenon has been reported before for a STAT1 variant (ref 38), where it became apparent when importin binding was lost due to deletion of the STAT N domain. There, it was accordingly explained to result from cytoplasmic accumulation of import-deficient P-STAT1, while concurrent nuclear export of the unphosphorylated STAT1 (U-STAT1) depleted the nuclear STAT1 pool. I would like to encourage the authors to do an initial exploration whether this mechanism applies also to STAT5, because they already used an N domain STAT deletion mutant in this work. In their SFig. 6, Ernst et al show that N domain-deletion the STAT5 likewise prevents cytokine-induced STAT5 nuclear accumulation. Cytoplasmic accumulation is not mentioned and apparent, which could be because only a single and relatively early time point (50 min) is shown, while cytoplasmic accumulation of the TAD-deleted variants is most prominent at later times. In my opinion, the inclusion of later time points could help clarify this aspect and would be a valuable addition to this work.

Minor point. The authors point out that STAT3 chimeras formed "punctate structures" upon cytokine treatment (Fig. 4D) and surmise they might result from "liquid-liquid phase separations". Such structures are readily apparent for the WT STAT3 (see also their Fig. 1C) and have been characterized by the Müller-Newen lab (JCS 2004) and others (Droescher et al, JBC, 2011). I think it might be useful to draw on the published literature to describe these structures instead of speculating.

Reviewer #2

(Remarks to the Author)
The study by Ernst et al. investigates nuclear–cytoplasmic shuttling of STAT5 using state-of-the-art live-cell imaging. Through an extensive panel of STAT5 deletion and point mutants, the authors identify a 12–amino acid region within the STAT5 transactivation domain (TAD) that mediates nuclear import in response to cytokine stimulation. They further show that two aspartic acid residues within this region (D754 and D758) are required, though not sufficient, for nuclear import of activated STAT5. Using the elegant CATCHFIRE approach, the authors also demonstrate that a single functional TAD within a phosphorylated STAT5 dimer is sufficient to drive nuclear import. Moreover, by employing chimeric TAD constructs between STAT3 and STAT5, they show that the STAT3 and STAT5 TADs have distinct roles in nucleocytoplasmic shuttling. Taken together, the authors identify a novel mechanism of nuclear import for STAT5 that differs from those of other STAT proteins and adds an important new dimension to STAT biology.
Overall, the findings are novel and of high interest to the field. The manuscript is clearly written and easy to follow. The experiments are well controlled, and the conclusions are well supported by the data.
Minor comments:
1.Please provide the sample sizes analyzed for each experiment and the number of independent biological replicates. For

imaging-based assays, consider specifying the number of cells/fields per condition and how many independent experiments were performed.
2.The statement that nuclear import “depends on tyrosine phosphorylation and dimerization of STAT5” should be slightly rephrased, since these events are coupled and cannot be fully disentangled in the current framework.

Version 1:

Reviewer comments:

Reviewer #1

(Remarks to the Author)
The authors have addressed my suggestions satisfactorily. In Fig 4C I noted that the pSTAT3 band is labelled pY694. This may need correction if it is an error.

Open Access This Peer Review File is licensed under a Creative Commons Attribution 4.0 International License, which permits use, sharing, adaptation, distribution and reproduction in any medium or format, as long as you give appropriate credit to the original author(s) and the source, provide a link to the Creative Commons license, and indicate if changes were made.
In cases where reviewers are anonymous, credit should be given to 'Anonymous Referee' and the source.
The images or other third party material in this Peer Review File are included in the article’s Creative Commons license, unless indicated otherwise in a credit line to the material. If material is not included in the article’s Creative Commons license and your intended use is not permitted by statutory regulation or exceeds the permitted use, you will need to obtain permission directly from the copyright holder.
To view a copy of this license, visit https://creativecommons.org/licenses/by/4.0/

Prof. Dr. rer. nat. Gerhard Müller-Newen
Institut für Biochemie und Molekularbiologie
Universitätsklinikum AöR
RWTH Aachen University
Pauwelsstraße 30
52074 Aachen
Germany
phone: +49 241 80-88860
e-mail: mueller-newen@rwth-aachen.de
May 8, 2026

COMMSBIO-26-0684-T

Point-by-point response to the reviewers' comments

The authors would like to thank the reviewers and the editor for their careful evaluation of our manuscript and for the constructive comments and suggestions. We greatly appreciate the time and effort invested in reviewing our work, which have helped us to improve the clarity and quality of the manuscript.

We hope that the revisions meet the expectations of the reviewers and the editorial office and that the manuscript will now be suitable for publication in Communications Biology.

All changes in the manuscript are marked in red.

Reviewer #1

Ernst et al present a well-executed and noteworthy study on STAT5 nuclear import. The authors demonstrate, using the appropriate controls, that the STAT5 transactivation domain (TAD) is required for the cytokine-induced nuclear accumulation of activated STAT5. In fact, they identify two key amino acids, and they demonstrate that nuclear-import assistance by the TAD is not required for other STAT proteins and cannot be provided by the TAD of another STAT. The phenomenon manifests as accumulation of phosphorylated STAT5 (P-STAT5) in the cytoplasm upon cytokine treatment. To my knowledge, this is the first report of such behaviour for a full-length STAT protein; it is characterised thoroughly in this paper yet underlying molecular mechanisms (e.g. enhanced export and/or reduced import) remain opaque.

Of note, the same phenomenon has been reported before for a STAT1 variant (ref 38), where it became apparent when importin binding was lost due to deletion of the STAT N domain. There, it was accordingly explained to result from cytoplasmic accumulation of import-deficient P-STAT1, while concurrent nuclear export of the unphosphorylated STAT1 (U-STAT1) depleted the nuclear STAT1 pool. I would like to encourage the authors to do an initial exploration whether this mechanism applies also to STAT5, because they already used an N domain STAT deletion mutant in this work. In their SFig. 6, Ernst et al show that N domain-deletion the STAT5 likewise prevents cytokine-induced STAT5 nuclear accumulation. Cytoplasmic accumulation is not mentioned and apparent, which could be because only a single and relatively early time point (50 min) is shown, while cytoplasmic accumulation of the TAD-deleted variants is most prominent at later times. In my opinion, the inclusion of later time points could help clarify this aspect and would be a valuable addition to this work.

The requested experiment has been performed, and the Discussion (page 8, 2nd paragraph) and Suppl. Fig. 6 have been adapted accordingly.

Minor point. The authors point out that STAT3 chimeras formed "punctate structures" upon cytokine treatment (Fig. 4D) and surmise they might result from "liquid-liquid phase separations". Such structures are readily apparent for the WT STAT3 (see also their Fig. 1C) and have been characterized by the Müller-Newen lab (JCS 2004) and others (Droescher et al, JBC, 2011). I think it might be useful to draw on the published literature to describe these structures instead of speculating.

The new references have been added and the corresponding Results section has been adapted accordingly (page 6).

Reviewer #2

The study by Ernst et al. investigates nuclear–cytoplasmic shuttling of STAT5 using state-of-the-art live-cell imaging. Through an extensive panel of STAT5 deletion and point mutants, the authors identify a 12–amino acid region within the STAT5 transactivation domain (TAD) that mediates nuclear import in response to cytokine stimulation. They further show that two aspartic acid residues within this region (D754 and D758) are required, though not sufficient, for nuclear import of activated STAT5. Using the elegant CATCHFIRE approach, the authors also demonstrate that a single functional TAD within a phosphorylated STAT5 dimer is sufficient to drive nuclear import. Moreover, by employing chimeric TAD constructs between STAT3 and STAT5, they show that the STAT3 and STAT5 TADs have distinct roles in nucleocytoplasmic shuttling. Taken together, the authors identify a novel mechanism of nuclear import for STAT5 that differs from those of other STAT proteins and adds an important new dimension to STAT biology.

Overall, the findings are novel and of high interest to the field. The manuscript is clearly written and easy to follow. The experiments are well controlled, and the conclusions are well supported by the data.

Minor comments:

1.Please provide the sample sizes analyzed for each experiment and the number of independent biological replicates. For imaging-based assays, consider specifying the number of cells/fields per condition and how many independent experiments were performed.

Sample sizes and/or number of independent experiments are now given in the figure legends. A Statistics and Reproducibility section has been added to the Methods (page 12).

2.The statement that nuclear import "depends on tyrosine phosphorylation and dimerization of STAT5" should be slightly rephrased, since these events are coupled and cannot be fully disentangled in the current framework.

The statement has been rephrased (page 8, 1st paragraph).
